# Author Correction: Remote loop evolution reveals a complex biological function for chitinase enzymes beyond the active site

**DOI:** 10.1038/s41467-026-73658-0

**Published:** 2026-05-25

**Authors:** Dan Kozome, Adnan Sljoka, Paola Laurino

**Affiliations:** 1 Protein Engineering and Evolution Unit, Okinawa Institute of Science and Technology Graduate University (OIST), Okinawa, 904-0495 Japan; 2 Center for Advanced Intelligence Project, RIKEN, Tokyo, 103−0027 Japan; 3 Department of Chemistry, York University, Toronto, ON M3J 1P3 Canada; 4 Institute for Protein Research, Osaka University, Suita, Japan

**Keywords:** Hydrolases, Molecular biophysics

Correction to: *Nature Communications* 10.1038/s41467-024-47588-8, published online 15 April 2024

In the version of the article initially published, the MD simulations were conducted as described in the Methods for the experiments shown in Figs. 3B, 4F, and Supplementary Fig. 5. During data plotting, one of the four replicas for Anc5ΔLoop I_r3 was inadvertently exchanged with one replica of Anc4+Loop II_r3. The data have now been replotted using the correct replicas, resolving the discrepancy and ensuring consistency with the deposited Source Data. In addition, in Fig. 3B, the range for the mean RMSF of Anc4+Loop II was inadvertently shifted by one column. These plotting errors do not affect the interpretation of the figures or the overall conclusions of the study. Figs. 3B, 4F, and Supplementary Fig. 5 have been corrected in the HTML and PDF versions of the article.

