# Supplementary material for: Remote loop evolution reveals a complex biological function for chitinase enzymes beyond the active site
Source: Nat Commun. 2024 Apr 15;15:3227. doi: 10.1038/s41467-024-47588-8 (PMC11018821; doi:10.1038/s41467-024-47588-8)
Supplement: Supplementary file 1 — Supplementary Information [file 41467_2024_47588_MOESM1_ESM.pdf]

SUPPLEMENTARY INFORMATION FOR

Beyond the Active Site: Remote loop evolution reveals a new complex biological function for chitinase enzymes

Dan Kozome[1], Adnan Sljoka[2,3], Paola Laurino[1,4]*

[1] Protein Engineering and Evolution Unit, Okinawa Institute of Science and Technology Graduate University (OIST), Okinawa 904-0495, Japan.

[2] Center for Advanced Intelligence Project, RIKEN, Tokyo 103-0027, Japan

[3] Department of Chemistry, York University, Toronto, ON M3J 1P3, Canada

[4] Institute for Protein Research, Osaka University, Suita, Japan

Corresponding author: Paola Laurino

Email: paola.laurino@oist.jp

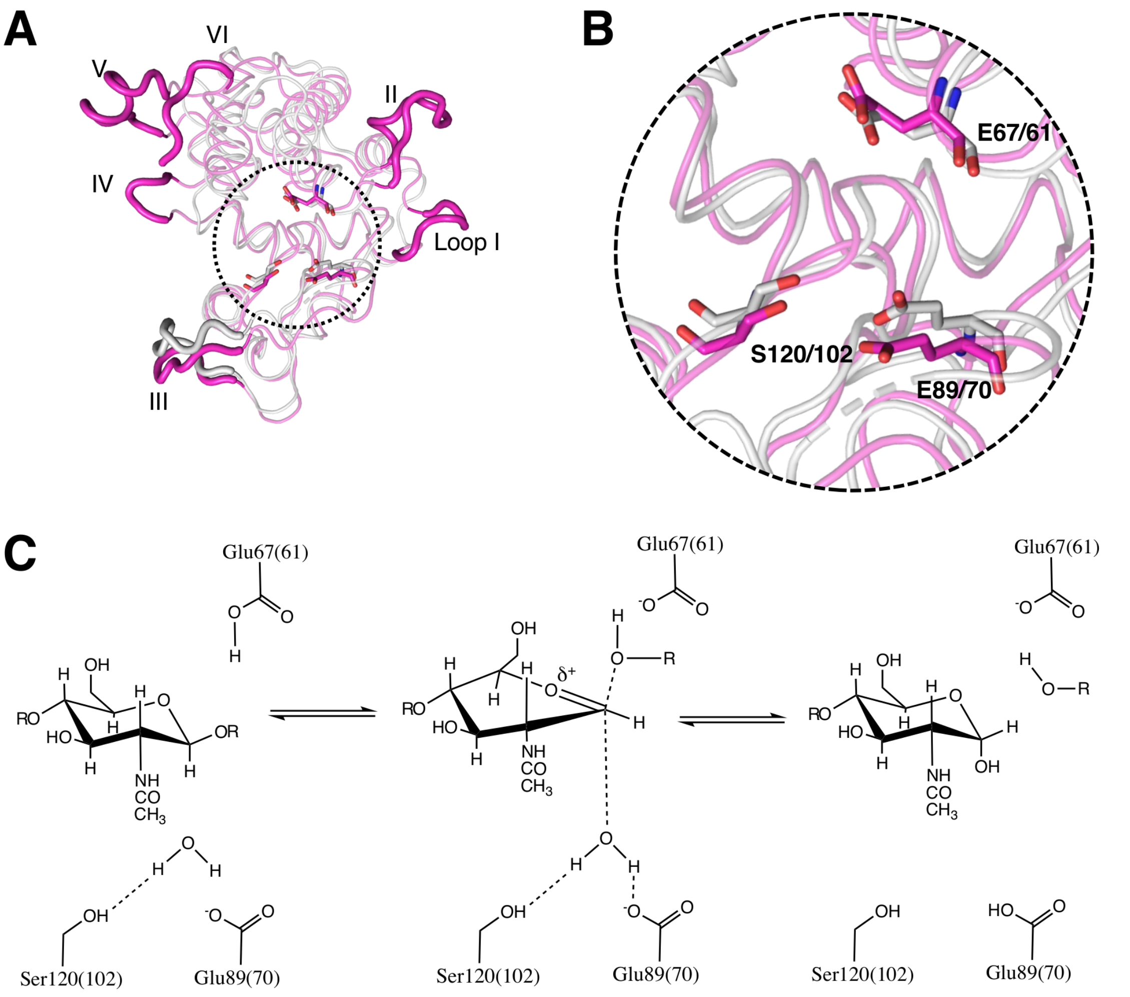

Supplementary Figure 1. Conserved catalytic resides and the catalytic mechanism of GH19 chitinase.

A, Superimposition of loopless (PDB ID:3WH1 with modification of replacing Ala 61 with Glu) and loopful (PDB ID:4J0L with modification of replacing Gln 67 with Glu) GH19 chitinases are shown respectively in gray and magenta. The dashed circle indicates the catalytic center. Highlighted in stick the catalytic residues. B, The enlarged view of the catalytic center shown in A. Two glutamate residues and one serine residue are shown in stick. C, The catalytic mechanism of hydrolysis of beta-1,4-glycosidic linkage proposed by Brameld and Goddard. The numbers of the two glutamate residues and one

serine residue are according to loopful GH19; those in parentheses are according to loopless GH19.

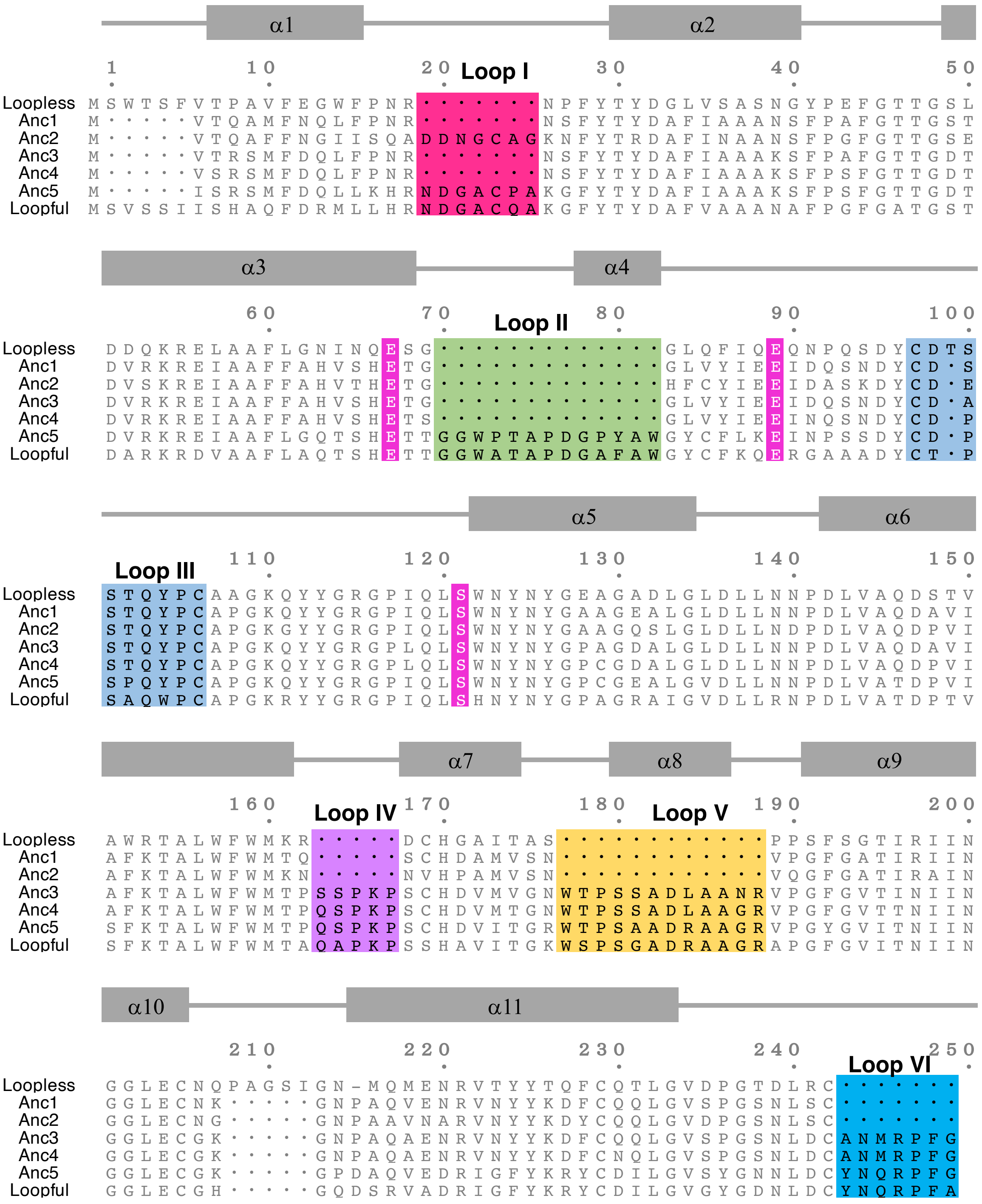
α1
α2
1 10 20 30 40 50
Loop I
Loopless MSWTSFVTPAVFEGWFPNR·······NPFYTYDGLVSASNGYPEFGTTGSL
Anc1 M·····VTQAMFNQLFPNR·······NSFYTYDAFIAAANSFPAFGTTGST
Anc2 M·····VTQAFFNGIISQADDNGCAGKNFYTRDAFINAANSFPGFGTTGSE
Anc3 M·····VTRSMFDQLFPNR·······NSFYTYDAFIAAAKSFPAFGTTGDT
Anc4 M·····VSRSMFDQLFPNR·······NSFYTYDAFIAAAKSFPSFGTTGDT
Anc5 M·····ISRSMFDQLLKHRNDGACPAKGFYTYDAFIAAAKSFPSFGTTGDT
Loopful MSVSSIISHAQFDRMLLHRNDGACQAKGFYTYDAFVAAANAFPGFGATGST
α3
α4
60 70 80 90 100
Loop II
Loopless DDQKRELAAFLGNINQESG·············GLQFIQEQNPQSDYCDTS
Anc1 DVRKREIAAFFAHVSHETG·············GLVYIEEIDQSNDYCD·S
Anc2 DVSKREIAAFFAHVTHETG·············HFCYIEEIDASKDYCD·E
Anc3 DVRKREIAAFFAHVSHETG·············GLVYIEEIDQSNDYCD·A
Anc4 DVRKREIAAFFAHVSHETS·············GLVYIEEINQSNDYCD·P
Anc5 DVRKREIAAFLGQTSHETTGGWPTAPDGPYAWGYCFLKEINPSSDYCD·P
Loopful DARKRDVAAFLAQTSHETTGGWATAPDGAFAWGYCFKQERGAAADYCT·P
α5
α6
110 120 130 140 150
Loop III
Loopless STQYPCAAGKQYYGRGPIQLSWNYNYGEAGADLGLDLLNNPDLVAQDSTV
Anc1 STQYPCAPGKQYYGRGPIQLSWNYNYGAAGEALGLDLLNNPDLVAQDAVI
Anc2 STQYPCAPGKGYYGRGPIQLSWNYNYGAAGQSLGLDLLNDPDLVAQDPVI
Anc3 STQYPCAPGKQYYGRGPLQLSWNYNYGPAGDALGLDLLNNPDLVAQDAVI
Anc4 STQYPCAPGKQYYGRGPLQLSWNYNYGPCGDALGLDLLNNPDLVAQDPVI
Anc5 SPQYPCAPGKQYYGRGPIQLSWNYNYGPCGEALGVDLLNNPDLVATDPVI
Loopful SAQWPCAPGKRYYGRGPIQLSHNYNYGPAGRAIGVDLLRNPDLVATDPTV
α7
α8
α9
160 170 180 190 200
Loop IV
Loop V
Loopless AWRTALWFWMKR·····DCHGAITAS············PPSFSGTIRIIN
Anc1 AFKTALWFWMTQ·····SCHDAMVSN············VPGFGATIRIIN
Anc2 AFKTALWFWMKN·····NVHPAMVSN············VQGFGATIRAIN
Anc3 AFKTALWFWMTPSSPKPSCHDVMVGNWTPSSADLAANRVPGFGVTINIIN
Anc4 AFKTALWFWMTPQSPKPSCHDVMTGNWTPSSADLAAGRVPGFGVTTNIIN
Anc5 SFKTALWFWMTPQSPKPSCHDVITGRWTPSAADRAAGRVPGYGVITNIIN
Loopful SFKTALWFWMTAQAPKPSSHAVITGKWSPSGADRAAGRAPGFGVITNIIN
α10
α11
210 220 230 240 250
Loop VI
Loopless GGLECNQPAGSIGN-MQMENRVTYYTQFCQTLGVDPGTDLRC·······
Anc1 GGLECNK·····GNPAQVENRVNYYKDFCQQLGVSPGSNLSC·······
Anc2 GGLECNG·····GNPAAVNARVNYYKDYCQQLGVDPGSNLSC·······
Anc3 GGLECGK·····GNPAQAENRVNYYKDFCQQLGVSPGSNLDCANMRPFG
Anc4 GGLECGK·····GNPAQAENRVNYYKDFCNQLGVSPGSNLDCANMRPFG
Anc5 GGLECGK·····GPDAQVEDRIGFYKRYCDILGVSYGNNLDCYNQRPFG
Loopful GGLECGH·····GQDSRVADRIGFYKRYCDILGVGYGDNLDCYNQRPFA

Supplementart Figure 2. A multiple sequence alignment of two extant GH19 chitinases and five inferred ancestral proteins.

Multiple sequence alignment was generated by MAFFT and Aliview. Residue numbering and the schematic representation of secondary structures are based on loopful type GH19 chitinase from *Secale cereale* (Uniprot: Q9FRV0; residues 24-266). Loopless indicates the sequence of GH19 chitinase from *Gemmabryum coronatum* (Uniprot: A9ZSX9; residues 25-228). They differ in loop regions, named I to VI highlighted red, green, light blue, purple, orange, and cyan, respectively. Two glutamate residues and one serine residue are shown in magenta.

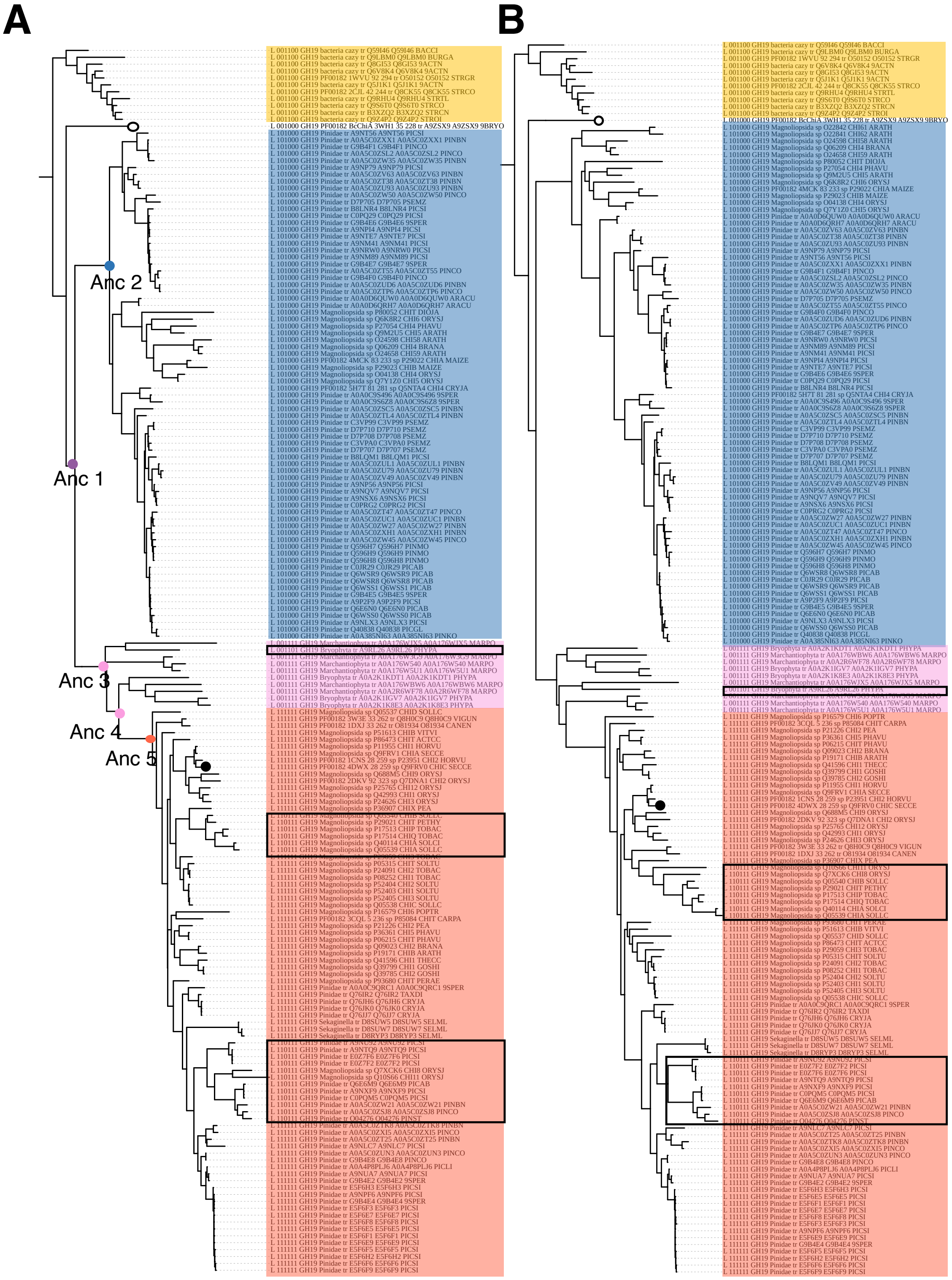
A
B
Anc 1
Anc 2
Anc 3
Anc 4
Anc 5

Supplementary Figure 3. Phylogenetic tree of GH19 chitinase constructed with whole sequences of GH19 catalytic domain (A) and sequences without six loop regions (B).

179 GH19 chitinase sequences containing 168 plants GH19 chitinases and 11 bacterial GH19 chitinases as an outgroup (highlighted in yellow) were used to construct maximum likelihood phylogenetic trees via IQ-TREE using WAG+F+I+G4 model. Sequences with 2, 4, and 6 loops are highlighted in blue, pink, and red, respectively. Open squares indicate the sequences with one loop deletion. The five ancestral nodes that were characterized in this study are labeled and colored according to the number of loop regions (purple, one loop; blue, two loops; pink, four loops; red, six loops).

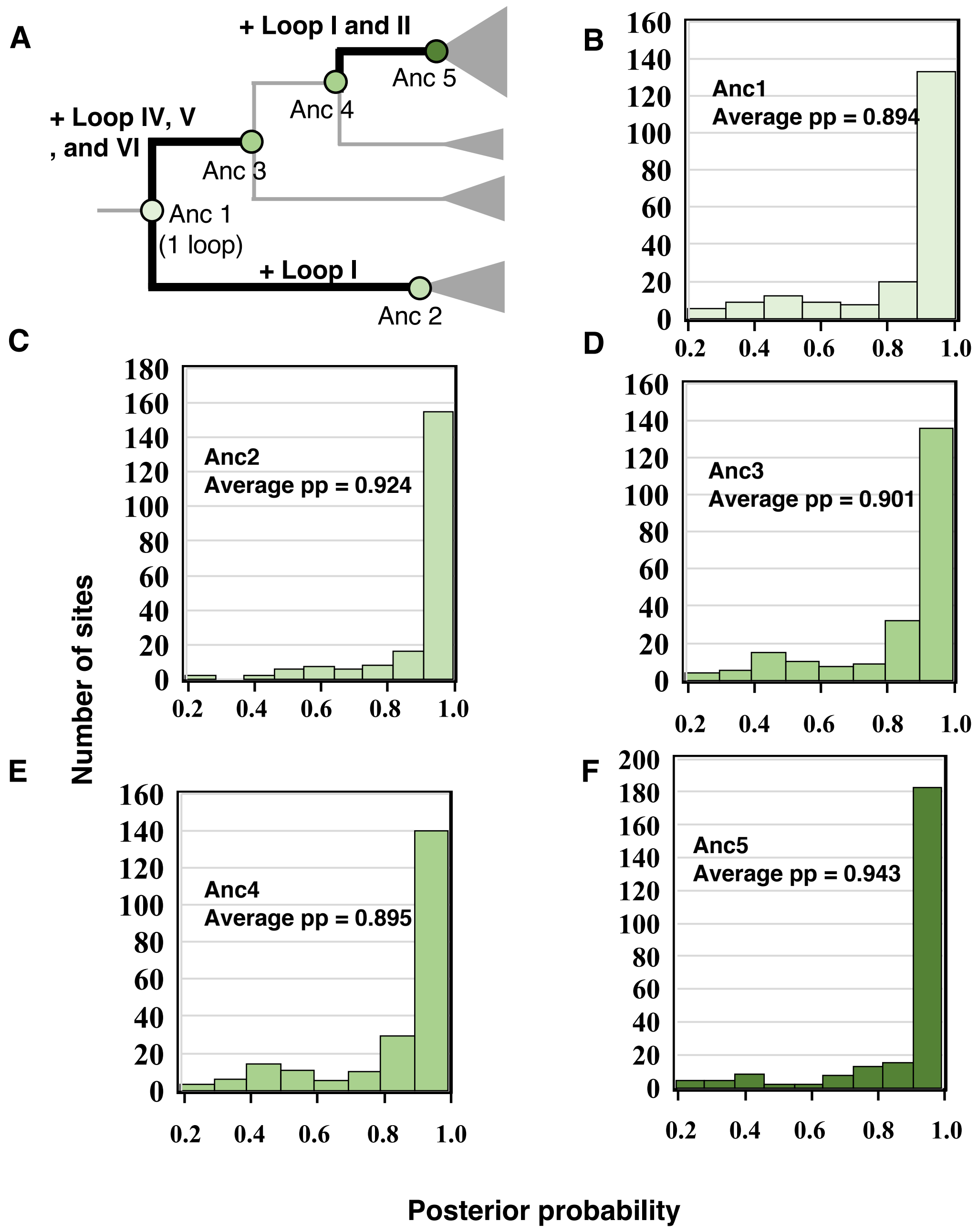

Supplementary Figure 4. Posterior probabilities for ancestral protein sequences.

A, Schematic representation of a phylogenetic tree of GH19 chitinase.

B-F, Distribution of posterior probabilities (pp) of the maximum a posteriori state at each amino acid site in the inferred ancestral sequences characterized in this study.

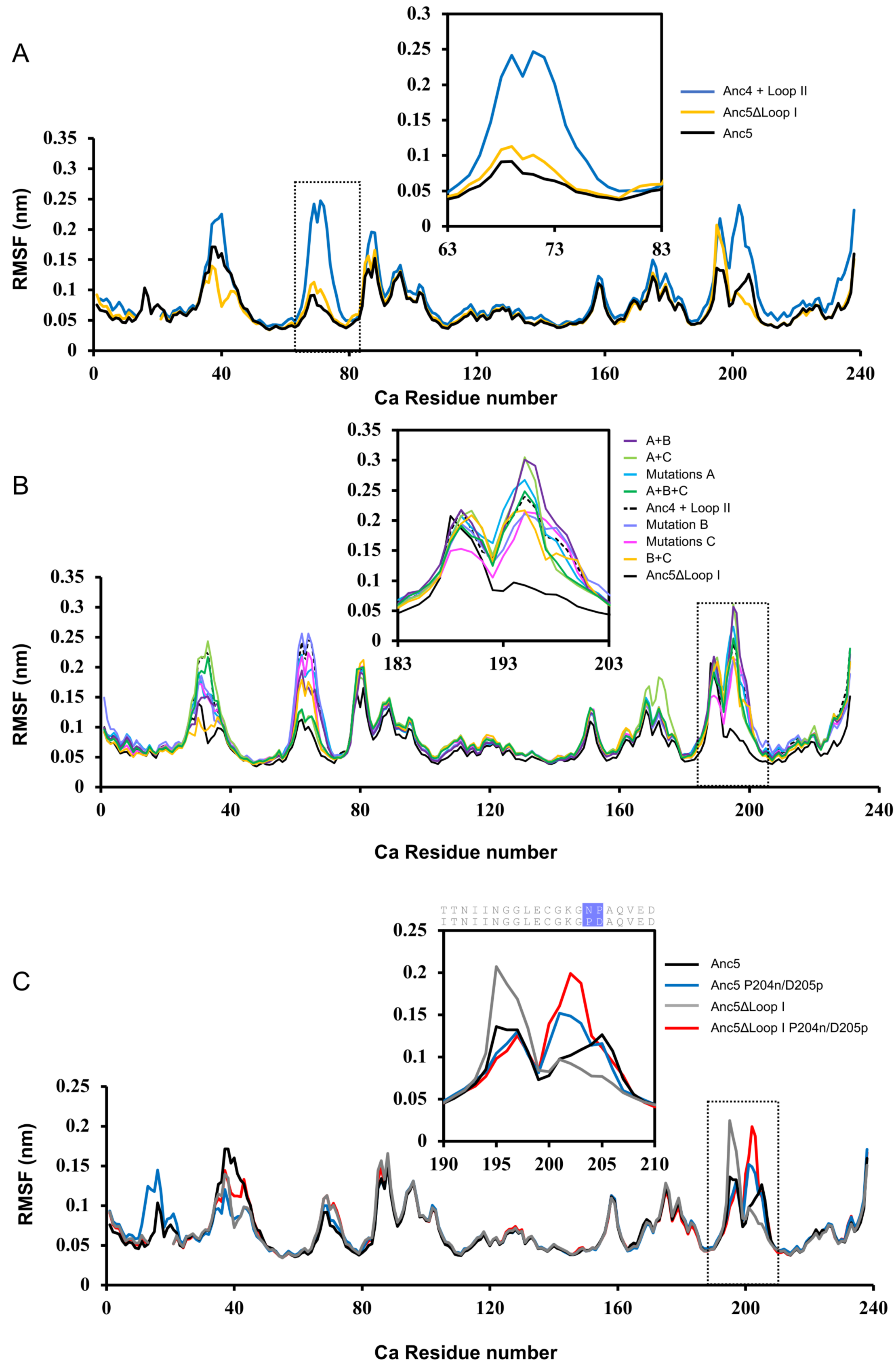
A
Anc4 + Loop II
Anc5ΔLoop I
Anc5
RMSF (nm)
Ca Residue number
B
A+B
A+C
Mutations A
A+B+C
Anc4 + Loop II
Mutation B
Mutations C
B+C
Anc5ΔLoop I
RMSF (nm)
Ca Residue number
C
TTNIINGGLECGKGNPAQVED
ITNIINGGLECGKGPDAQVED
Anc5
Anc5 P204n/D205p
Anc5ΔLoop I
Anc5ΔLoop I P204n/D205p
RMSF (nm)
Ca Residue number

Supplementary Figure 5. Mutation effects on loops mobilities.

A), Plots of the RMSF of Cα of each residue in Anc4 + Loop II (blue), Anc5ΔLoop I (orange), and Anc5 (gray). In the enlargement, it showed reduced mobility of the loop II regions (residue number 66-78) in Anc5ΔLoop I and Anc5 compared to Anc4 + Loop I.

B), Plots of the RMSF of Cα of each residue in Anc5ΔLoop I (black), Anc4 + Loop II (dashed-black), and Anc4 + Loop II mutants. In the enlargement, it showed the movement in a loop region (positions 192 – 201), where the reduced mobility observed in Anc5ΔLoop I compared to Anc4 + Loop II. Introducing substitutions that stabilize loop II and enhance antifungal activity did not stabilize this loop region (positions 192 – 201).

C), Plots of the RMSF of Cα of each residue in Anc5 (black), Anc5ΔLoop I (gray), and their mutants containing Anc4 state (P204n/D205p) in a loop region (positions 199-208, the residue number corresponds to Anc5 state) described in B).

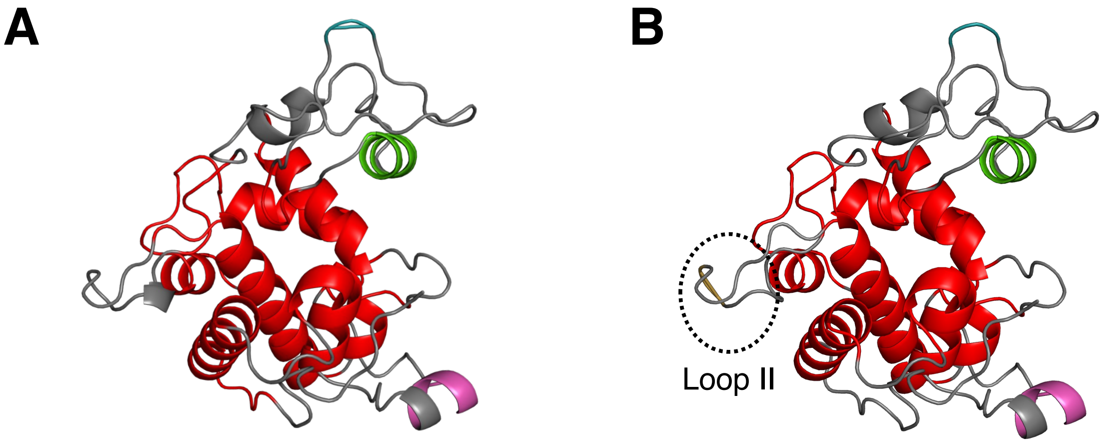

Anc4 + Loop II

Anc5ΔLoop I

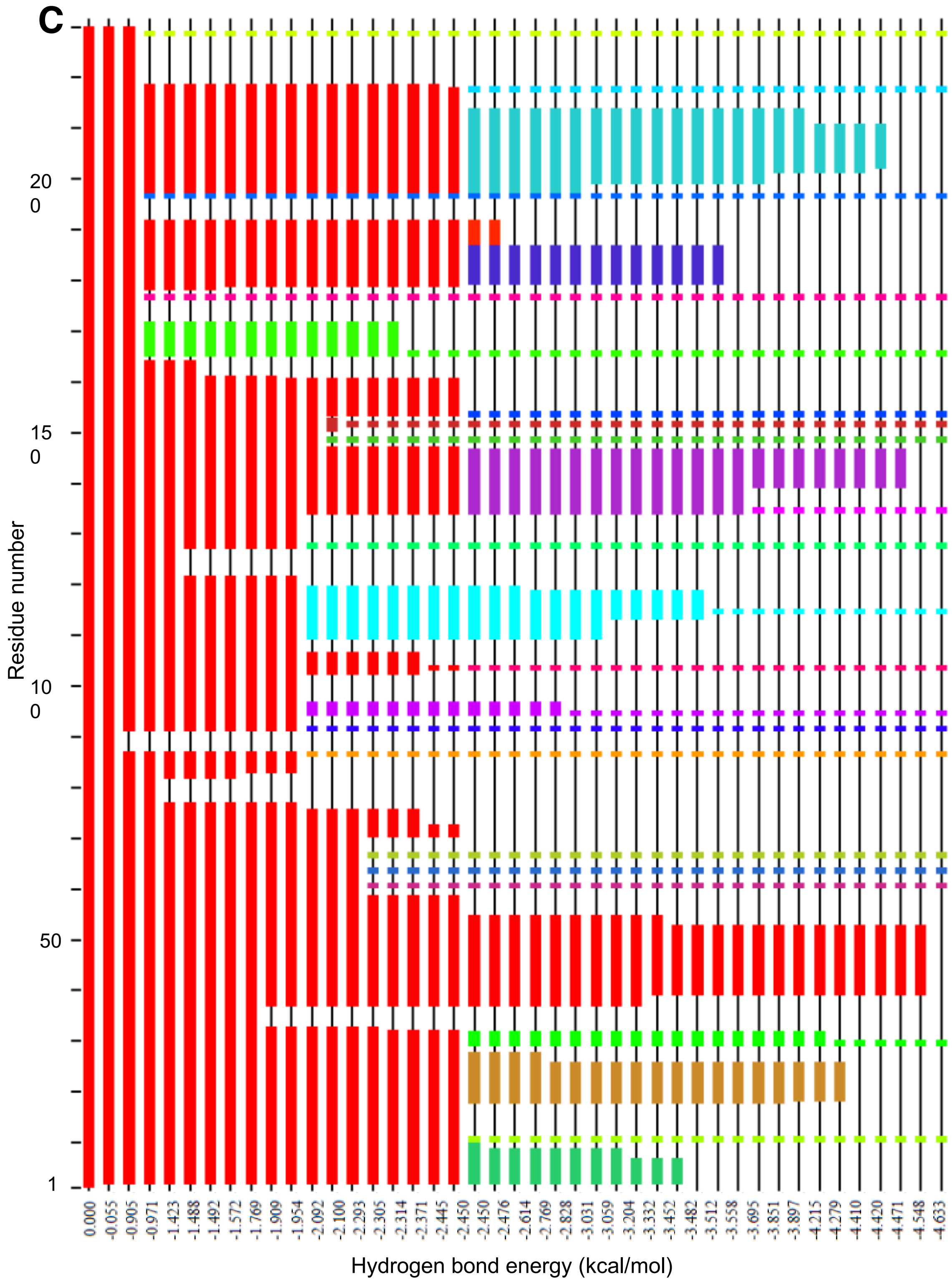

C
Residue number
20
0
15
0
10
0
50
1
0.000
-0.055
-0.905
-0.971
-1.423
-1.488
-1.492
-1.572
-1.769
-1.909
-1.954
-2.092
-2.100
-2.293
-2.305
-2.314
-2.371
-2.445
-2.450
-2.450
-2.476
-2.614
-2.769
-2.828
-3.031
-3.059
-3.204
-3.332
-3.452
-3.482
-3.512
-3.558
-3.695
-3.851
-3.897
-4.215
-4.279
-4.410
-4.420
-4.471
-4.548
-4.633
Hydrogen bond energy (kcal/mol)

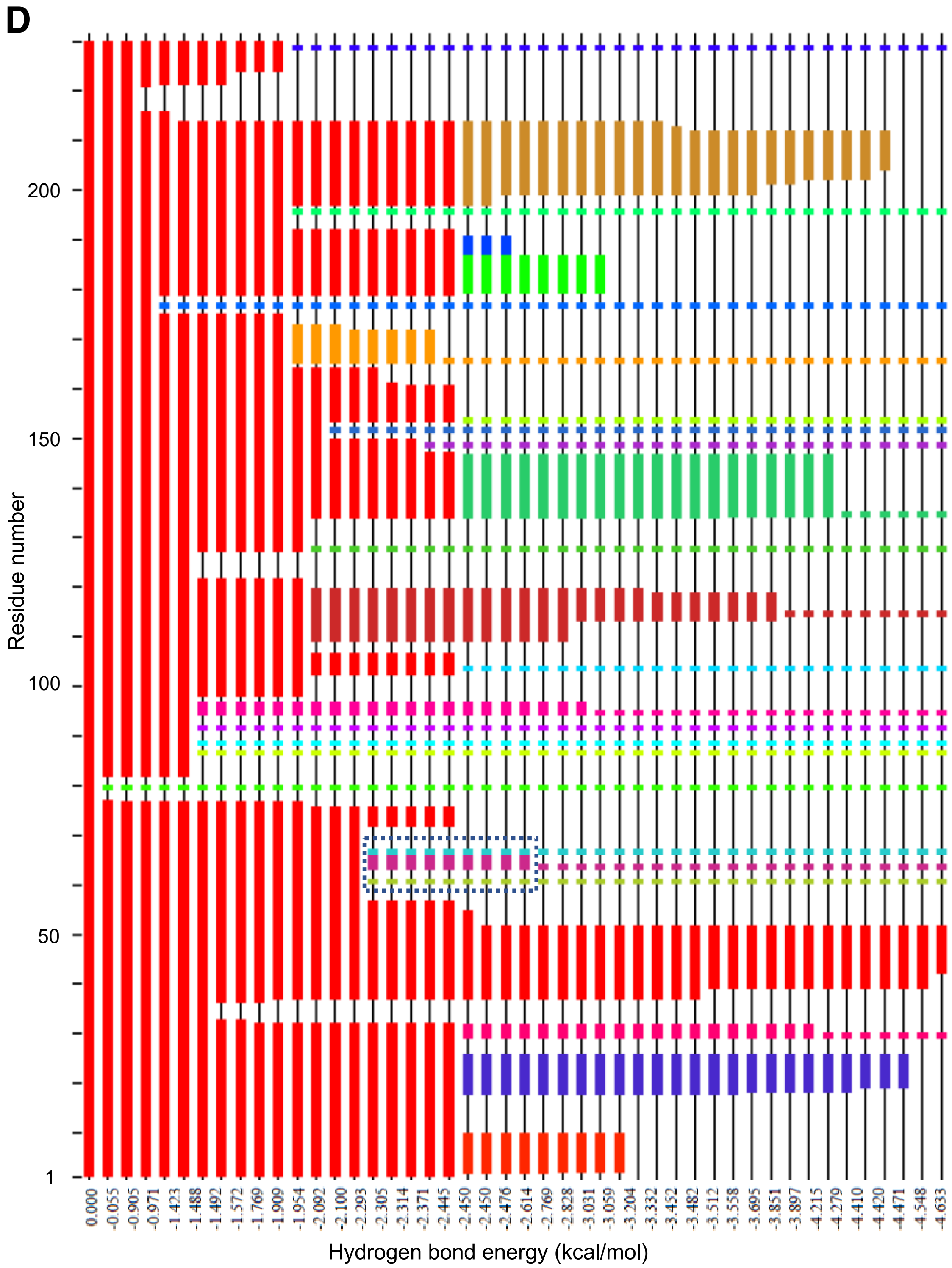

Supplementary Figure 6. Computational analysis of long-range communication and rigidity.

A and B, Left, Rigid cluster decomposition using program FIRST of Anc4 + Loop II and Anc5ΔLoop I at hydrogen bond energy strength cutoff -2.3 kcal/mol are mapped on 3D structures. Distinct rigid clusters are designated by different colored regions, while flexible regions are shown in gray. Red represents the largest dominant rigid cluster. Anc5ΔLoop I has an additional rigid cluster in loop II highlighted with a dashed circle. Right, Rigidity profile of Anc4 + Loop II and Anc5ΔLoopI indicated with a hydrogen bond dilution plot with program FIRST. The horizontal axis depicts the residue numbers. The vertical axis represents the current hydrogen bond energy cutoff in kcal/mol. Flexible regions are indicated as black horizontal thin lines and distinct rigid clusters are indicated by various colored blocks with red being the largest rigid cluster. As weak hydrogen bonds are removed, clusters become fragmented, and structure becomes increasingly flexible. Dashed square indicates the rigid cluster within loop II that is observed only in Anc5ΔLoop I.

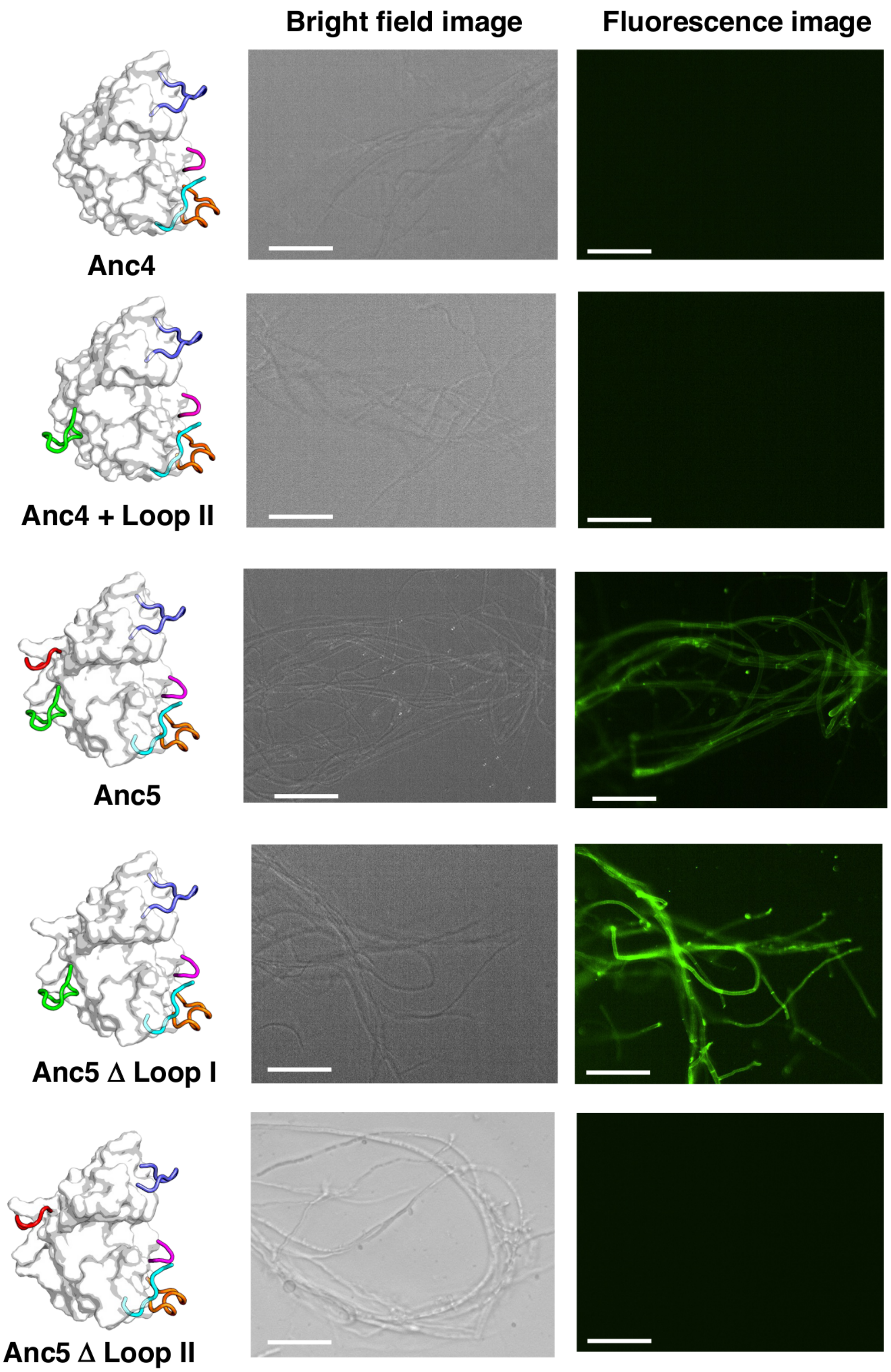
Bright field image
Fluorescence image
Anc4
Anc4 + Loop II
Anc5
Anc5 Δ Loop I
Anc5 Δ Loop II

Supplementary Figure 7. Fluorescence microscopy images of fluorophore labelled Anc4 and Anc5 and their mutants on the surface of fungal hyphae revealed that antifungal activity requires the binding activity to the fungal hyphae.

Each 50 μL of 2 μM Alexa Fluor 488-labelled protein samples are mixed with *T. longibrachiatum* hyphae in 20 mM sodium phosphate buffer, pH 7.4 at 25 °C.

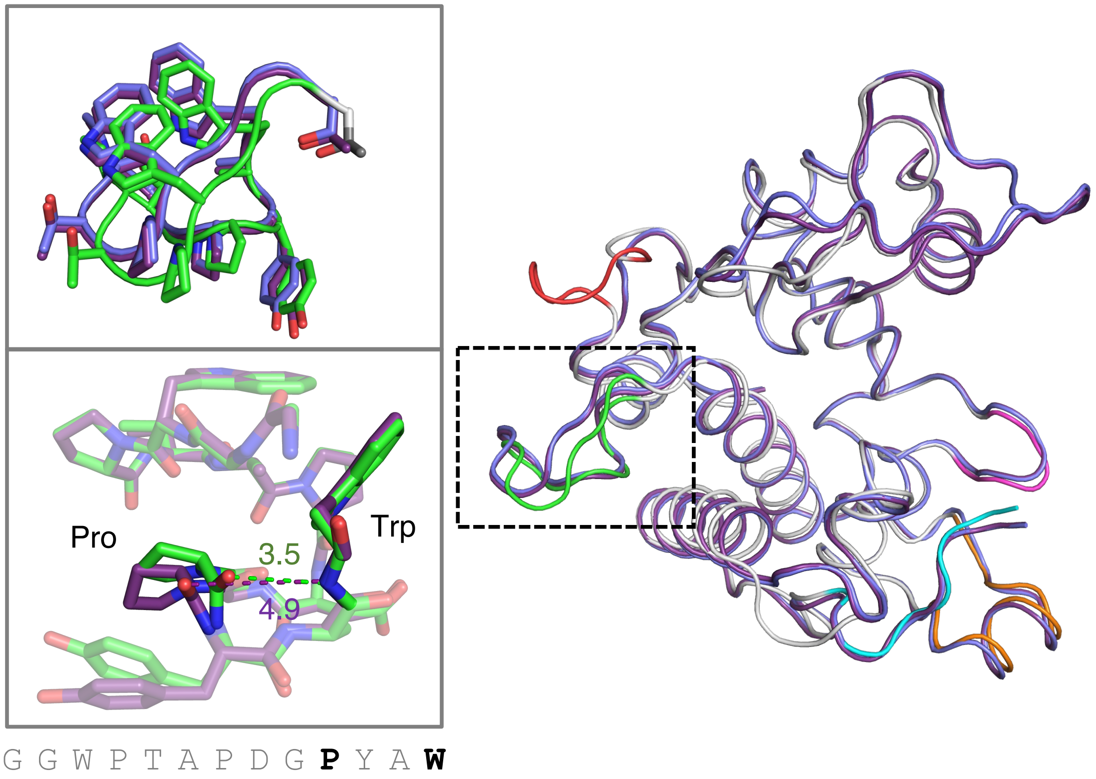

Supplementary Figure 8. Structural comparison of Anc5, Anc4+Loop II +A, and Anc4+Loop II +A+B+C.

Superimpositions of Anc5, Anc4+Loop II +A, and Anc4+Loop II +A+B+C showed that their structures were almost identical with a slight shift in loop II regiosn squared in a dashed line. Cartoon_loop representations of Anc4+Loop II +A and Anc4+Loop II +A+B+C are shown in slate and purple, respectively. Cartoon_loop representations of Anc5 is shown in white with loop regions I-VI highlighted in red, green, slate, magenta, orange, cyan, respectively. In the bottom enlargement, the pairwise structural alignment in loop II regions of Anc5 and Anc4+Loop II +A+B+C revealed that the hydrogen bonding interaction between Pro75 and Trp78 observed in Anc5 (green) was diminished in Anc4+Loop II +A+B+C (purple).

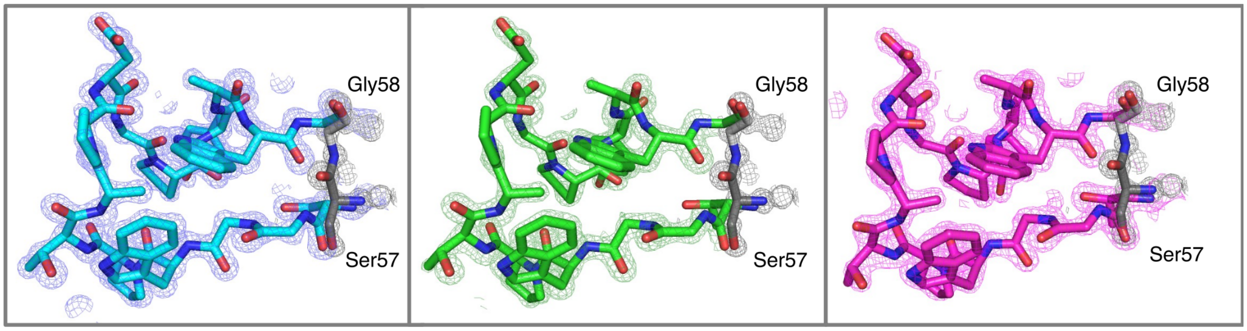

Anc4 +Loop II +p12K/n13H Anc4+LoopII+p12K/n13H/s58T/n193G/y194F/d197R Anc5

Supplementary Figure 9. Structures of the Loop II regions aligned with loop II inserted residues in Anc4 (Ser57 and Gly58). |2Fo-Fc| electron density maps contoured at 2 σ were shown in gray, blue, green, and magenta for Anc4 (8HNE), Anc4+Loop II+p12K/n13H (8X2V), Anc4+LoopII+p12K/n13H/s58T/n193G/y194F/d197R (8X2W), and Anc5 (8HNF), respectively.

Supplementary Table 1 Primers used in this study

Primers	Sequence 5' to 3'
T7	TAATACGACTCACTATAGGG
T7_terminator	GCTAGTTATTGCTCAGCGG
Linear_vector_F	TAGTGAGTCGTATTAATTTCG
Linear_vector_R	TGAGCAATAACTAGCATAACCCCTTG
**Anc4**	
+Loop I_F	GCCCTTCGCAGGGCACGCGCCACTATTACGGTTCGGGAACAG
+Loop I_R	GGCGCGTGCCCTGCGAAGGGCTTTTACACGTATGACGCG
+Loop II_F	CCACGCGTAAGGGCCATCCGGTGCGGTCGGCCATCCACCACTAGTTTCGTGACTCAC
+Loop II_R	GGTGGATGGCCGACCGCACCGGATGGCCCTTACGCGTGGGGCTTGGTATATATTGAGGAAATTAATC
E57Q_F	ACTAGTTTGGTGACTCACATGTGC
E57Q_R	AGTCACCAAACTAGTGGCTTGG
**Anc4+LoopII**	
E57Q_R	AGTCACCAAACTAGTGGTGGATGGC
p12K/n13H_F	ATTACGGTGTTTGAACAGTTGGTCGAACAT
p12K/n13H_R	CAACTGTTCAAACACCGTAATAGTTTTTAC
s58T_F	TCCACCAGTAGTTTCGTGACTCACATG
s58T_R	GAAACTACTGGTGGATGGCCGACCGCAC
n193G/y194F/d197R_F	GAATCTTTTATAAAATCCCACACGATTCTC
n193G/y194F/d197R_R	GTGGGATTTTATAAAAGATTCTGCAATCAG
**Anc5**	
ΔLoop I_F	TGTAGAAGTCGTTGCGGTGTTTCAA
ΔLoop I_R	CAACGACTTCTACACATACGATGCG
ΔLoop II_F	AGTAGCCTGTAGTCTCATGACTCGTC
ΔLoop I_R	GACTACAGGCTACTGTTTTCTGAAAGAG
E64Q_F	TGTAGTCTGATGACTCGTCTGACC
E64Q_R	AGTCATCAGACTACAGGTGGATGGC
P204n/D205p_F	TTGTGCCGGGTTTCCTTTCCCGCACTC
P204n/D205p_R	GGGAAAGGAAACCCGGCACAAGTAGAGGAC
**Anc5ΔLoopII**	
E64Q_R	AGTCATCAGACTACAGGCTACTGTTTTC

Supplementary Table 2 Experimental Details for crystallization of Anc4 and Anc5

**Structure**	Anc4	Anc5
**PDB code**	8HNE	8HNF
**Buffer**	5 mM Tris-HCl pH 8.0	5 mM Tris-HCl pH 8.0
**Crystallization**	Hanging drop, 1 µL of Anc4 (10 mg/mL) + 1 µL of 15% (w/v) PEG mono ether 2,000, 0.1 M MES pH 6.0	Hanging drop, 1 µL of Anc5 (5.67 mg/mL) + 1 µL of 27% (w/v) PEG mono ether 2,000, 0.1 M MES pH 6.0
**Cryoprotectant**	40% (w/v) PEG monomethyl ether 2,000, 0.1 M MES pH 6.0	40% (w/v) PEG monomethyl ether 2,000, 0.1 M MES pH 6.0
**Wavelength (Å)**	1.000	1.000
**Beamline**	SPring-8 BL45XU	SPring-8 BL41XU
**Data processing**	KAMO, XDS, XSCALE	XDS, Aimless
**MR model**	AlphaFold2	AlphaFold2
**Refinement**	The model building was performed with *Coot*. The model structure was refined by *Coot* and Phaser.	The model building was performed with *Coot*. The model structure was refined by *Coot* and Phaser.

Supplementary Table 3 Experimental Details for crystallization of Anc4+Loop II mutants

**Structure**<br>**PDB code**	Anc4+Loop II+p12K/n13H	Anc4+Loop II+p12K/n13H/s58T/n193G/y194F/d197R
**Buffer**	10 mM Sodium acetate,<br>150 mM NaCl pH 5.0	10 mM Sodium acetate,<br>150 mM NaCl pH 5.0
**Crystallization**	Sitting drop, 1 μL of Anc4+Loop II+p12K/n13H (6.00 mg/mL) + 1 μL of 25% (w/v) polyethylene glycol 3,350, 0.1 M Bis-Tris, pH 6.5	Sitting drop, 1 μL of Anc4+Loop II+p12K/n13H/s58T/n193G/y194F/d197R (3.80 mg/mL) + 1 μL of 25% (w/v) polyethylene glycol 3,350, 0.1 M Bis-Tris, pH 6.5
**Cryoprotectant**	30% (w/v) polyethylene glycol 3,350, 0.1 M Bis-Tris, pH 6.5	30% (w/v) polyethylene glycol 3,350, 0.1 M Bis-Tris, pH 6.5
**Wavelength (Å)**	1.000	1.000
**Beamline**	SPring-8 BL32XU	SPring-8 BL32XU
**Data processing**	KAMO, XDS, XSCALE	KAMO, XDS, XSCALE
**MR model**	AlphaFold2	AlphaFold2
**Refinement**	The model building was performed with *Coot*. The model structure was refined by *Coot* and Phaser.	The model building was performed with *Coot*. The model structure was refined by *Coot* and Phaser.

Supplementary Table 4 Data collection and refinement statistics for Anc4 and Anc5

Structure	Anc4	Anc5
PDB code	8HNE	8HNF
**Data collection**		
Space group	$P2_12_12_1$	$P12_11$
Cell dimensions		
a, b, c (Å)	32.29, 76.15, 79.18	36.89, 73.43, 39.28
α, β, γ (°)	90.00, 90.00, 90.00	90.00, 99.965, 90.00
Resolution (Å)	39.47 – 1.13 (1.17-1.13)*	38.69 – 1.571 (1.627-1.571)
R_{merge}	0.2433 (0.325)	0.0658 (0.484)
$I / \sigma I$	4.14 (2.54)	10.36 (1.18)
Completeness (%)	99.62 (96.37)	99.54 (96.92)
Redundancy	2.0 (2.0)	2.0 (2.0)
**Refinement**		
Resolution (Å)	39.47 – 1.13	38.69 – 1.57
No. reflections	73261 (6976)	28613 (2773)
R_{work} / R_{free} (%)	24.07 / 25.05	17.60 / 20.21
No. atoms		
Protein	1700	1881
Ligand/ion	91	66
Water	214	86
B-factors		
Protein	8.82	25.15
Ligand/ion	14.53	38.25
Water	17.40	30.73
R.m.s. deviations		
Bond lengths (Å)	0.007	0.010
Bond angles (°)	1.09	1.08

*Values in parentheses are for highest-resolution shell.

Supplementary Table 5 Data collection and refinement statistics for Anc4+Loop II mutants

Structure<br>PDB code	Anc4+LoopII+p12K/n13H<br>(8X2V)	Anc4+LoopII+p12K/n13H/s58T/n193G/y194F/d197R<br>(8X2W)
**Data collection**		
Space group	$P22_12_1$	$P22_12_1$
Cell dimensions		
a, *b*, *c* (Å)	37.29, 69.83, 70.27	37.32, 69.59, 70.22
α, β, γ (°)	90.00, 90.00, 90.00	90.00, 90.00, 90.00
Resolution (Å)	49.53 – 1.29 (1.336-1.29)*	49.43 – 1.40 (1.45-1.40)
R_{merge}	18.6 (162.2)	234.1 (-99.9)
$I / \sigma I$	12.87 (2.13)	7.00 (0.00)
Completeness (%)	100.0 (100.0)	99.99 (100.0)
Redundancy	33.1 (26.4)	55.3 (54.2)
**Refinement**		
Resolution (Å)	39.47 – 1.13	38.69 – 1.57
No. reflections	47013 (4640)	36817 (3631)
R_{work} / R_{free} (%)	17.1 / 18.6	18.64 / 20.41
No. atoms		
Protein	1818	1799
Ligand/ion	0	0
Water	134	118
B-factors		
Protein	14.60	23.24
Ligand/ion	0	0
Water	18.95	28.35
R.m.s. deviations		
Bond lengths (Å)	0.007	0.009
Bond angles (°)	0.96	1.06

*Values in parentheses are for highest-resolution shell.

Supplementary Table 6 Effects of mutations that destabilize a loop region on enzymatic activity.

variants	Hydrolytic activity (U*/mol) $\times 10^9$	Antifungal activity (IC_{50}, μM)
Anc5	0.85	16.9 ± 1.4
Anc5ΔLoopI	1.28	17.2 ± 1.9
Anc5 P204n/D205p	0.92	15.4 ± 0.2
Anc5ΔLoopI P204n/D205p	1.32	19.4 ± 2.7

One unit of activity is defined as the enzyme activity that produced one μmol of GlcNAc per minute at 37°C.

Supplementary Table 7 Systems used for MD simulations.

Proteins	Total atoms	Water	Ions	Box sixe (nm)								
Anc4	159156	51960	5 Na^+	13.08	13.08	9.25	0.00	0.00	0.00	0.00	6.54	6.54
Anc4+Loop II	156054	50872	6 Na^+	13.01	13.01	9.20	0.00	0.00	0.00	0.00	6.50	6.50
Anc5	166729	54380	Na^+	13.30	13.30	9.41	0.00	0.00	0.00	0.00	6.65	6.65
Anc5ΔLoop I	155306	50606	2 Na^+	13.00	13.00	9.18	0.00	0.00	0.00	0.00	6.50	6.50
Anc4+Loop II+A	156199	50917	5 Na^+	13.02	13.02	9.20	0.00	0.00	0.00	0.00	6.51	6.51
Anc4+Loop II+B	156120	50893	6 Na^+	13.01	13.01	9.20	0.00	0.00	0.00	0.00	6.50	6.50
Anc4+Loop II+C	158165	51575	4 Na^+	13.06	13.06	9.24	0.00	0.00	0.00	0.00	6.53	6.53
Anc4+Loop II+A+B	156115	50888	5 Na^+	13.00	13.00	9.18	0.00	0.00	0.00	0.00	6.50	6.50
Anc4+Loop II+A+C	156204	50918	3 Na^+	13.01	13.01	9.20	0.00	0.00	0.00	0.00	6.50	6.50
Anc4+Loop II+B+C	158147	51568	4 Na^+	13.06	13.06	9.24	0.00	0.00	0.00	0.00	6.53	6.53
Anc4+Loop II+A+B+C	155205	50584	3 Na^+	12.98	12.98	9.17	0.00	0.00	0.00	0.00	6.49	6.49
Anc5 P204n/D205p	165120	53849	Na^+	13.24	13.24	9.36	0.00	0.00	0.00	0.00	6.62	6.62
Anc5ΔLoop I P204n/D205p	155086	50530	0	12.98	12.98	9.18	0.00	0.00	0.00	0.00	6.49	6.49
