# Supplementary material for: Remote loop evolution reveals a complex biological function for chitinase enzymes beyond the active site
Source: Nat Commun. 2024 Apr 15;15:3227. doi: 10.1038/s41467-024-47588-8 (PMC11018821; doi:10.1038/s41467-024-47588-8)
Supplement: Supplementary file 2 — Reporting Summary [file 41467_2024_47588_MOESM2_ESM.pdf]

nature portfolio

Corresponding author(s): Paola Laurino

Last updated by author(s): Mar 26, 2024

Reporting Summary

Nature Portfolio wishes to improve the reproducibility of the work that we publish. This form provides structure for consistency and transparency in reporting. For further information on Nature Portfolio policies, see our Editorial Policies and the Editorial Policy Checklist.

Statistics

For all statistical analyses, confirm that the following items are present in the figure legend, table legend, main text, or Methods section.

n/a	Confirmed	
☐	☒	The exact sample size (*n*) for each experimental group/condition, given as a discrete number and unit of measurement
☒	☐	A statement on whether measurements were taken from distinct samples or whether the same sample was measured repeatedly
☒	☐	The statistical test(s) used AND whether they are one- or two-sided<br>*Only common tests should be described solely by name; describe more complex techniques in the Methods section.*
☒	☐	A description of all covariates tested
☒	☐	A description of any assumptions or corrections, such as tests of normality and adjustment for multiple comparisons
☐	☒	A full description of the statistical parameters including central tendency (e.g. means) or other basic estimates (e.g. regression coefficient) AND variation (e.g. standard deviation) or associated estimates of uncertainty (e.g. confidence intervals)
☒	☐	For null hypothesis testing, the test statistic (e.g. *F*, *t*, *r*) with confidence intervals, effect sizes, degrees of freedom and *P* value noted<br>*Give P values as exact values whenever suitable.*
☒	☐	For Bayesian analysis, information on the choice of priors and Markov chain Monte Carlo settings
☒	☐	For hierarchical and complex designs, identification of the appropriate level for tests and full reporting of outcomes
☒	☐	Estimates of effect sizes (e.g. Cohen's *d*, Pearson's *r*), indicating how they were calculated

Our web collection on statistics for biologists contains articles on many of the points above.

Software and code

Policy information about availability of computer code

Data collection	BlastP (ver. 2.6.0) is used for collecting GH19 chitinase homolog sequences from GenBank
Data analysis	MAFFT ver. 7 is used to align sequences.<br>Aliview ver.1.28 is used to edit multiple sequence alignment<br>IQ-TREE 1.6.12 is used for phylogenetic analysis and ancestral sequence reconstruction<br>GROMACS version 2020.1 is used for MD simulations<br>Phenix 1.20 is used for structure refinement<br>Coot ver. 0.8.9.2 was used for 3D-structure refinement

For manuscripts utilizing custom algorithms or software that are central to the research but not yet described in published literature, software must be made available to editors and reviewers. We strongly encourage code deposition in a community repository (e.g. GitHub). See the Nature Portfolio guidelines for submitting code & software for further information.

Data

Policy information about availability of data

All manuscripts must include a data availability statement. This statement should provide the following information, where applicable:

- Accession codes, unique identifiers, or web links for publicly available datasets
- A description of any restrictions on data availability
- For clinical datasets or third party data, please ensure that the statement adheres to our policy

Atomic coordinates and the experimental structure factors have been deposited in Protein Data Bank (https://www.rscb.org) with ID 3WH1, 4J0L, 8HNE, 8HNF, 8X2V, and 8X2W for loopless type GH19 chitinase from Gemmabryum coronatum, loopful type GH19 chitinase from Secale cereale, Anc4, Anc5, Anc4+LoopII+p12K/n13H, and Anc4+LoopII+p12K/n13H/s58T/n193G/y194F/d197R, respectively. Parameters, input files, output files from our MD simulations are available for download from Zenodo (https://zenodo.org) at https://zenodo.org/records/10730202. Source data are provided with this paper.

Research involving human participants, their data, or biological material

Policy information about studies with human participants or human data. See also policy information about sex, gender (identity/presentation), and sexual orientation and race, ethnicity and racism.

Reporting on sex and gender	n/a
Reporting on race, ethnicity, or other socially relevant groupings	n/a
Population characteristics	n/a
Recruitment	n/a
Ethics oversight	n/a

Note that full information on the approval of the study protocol must also be provided in the manuscript.

Field-specific reporting

Please select the one below that is the best fit for your research. If you are not sure, read the appropriate sections before making your selection.

[x] Life sciences [ ] Behavioural & social sciences [ ] Ecological, evolutionary & environmental sciences

For a reference copy of the document with all sections, see nature.com/documents/nr-reporting-summary-flat.pdf

Life sciences study design

All studies must disclose on these points even when the disclosure is negative.

Sample size	Not applicable.<br>In the context of this study focused on protein engineering, we employ biophysical methodologies to elucidate specific characteristics of proteins. Unlike studies that rely on statistical hypothesis testing to validate predefined hypotheses, such as those common in biomedical or animal behavior studies, our approach does not fit this context. Then, traditional statistical hypothesis testing is not applicable to our methodology. Instead, in our field, the crucial biophysical characterizations are performed repeatedly (three times in general) to have an estimation of the error associated to the determined value.
Data exclusions	No data have been exculded
Replication	Not applicable.<br>In the context of this study focused on protein engineering, we employ biophysical methodologies to elucidate specific characteristics of proteins. Unlike studies that rely on statistical hypothesis testing to validate predefined hypotheses, such as those common in biomedical or animal behavior studies, our approach does not fit this context. Then, traditional statistical hypothesis testing is not applicable to our methodology. Instead, in our field, the crucial biophysical characterizations are performed repeatedly (three times in general) to have an estimation of the error associated to the determined value.
Randomization	Not applicable.<br>In the context of this study focused on protein engineering, we employ biophysical methodologies to elucidate specific characteristics of proteins. Unlike studies that rely on statistical hypothesis testing to validate predefined hypotheses, such as those common in biomedical or animal behavior studies, our approach does not fit this context. Then, traditional statistical hypothesis testing is not applicable to our methodology. Instead, in our field, the crucial biophysical characterizations are performed repeatedly (three times in general) to have an estimation of the error associated to the determined value.
Blinding	Not applicable.<br>In the context of this study focused on protein engineering, we employ biophysical methodologies to elucidate specific characteristics of

proteins. Unlike studies that rely on statistical hypothesis testing to validate predefined hypotheses, such as those common in biomedical or animal behavior studies, our approach does not fit this context. Then, traditional statistical hypothesis testing is not applicable to our methodology. Instead, in our field, the crucial biophysical characterizations are performed repeatedly (three times in general) to have an estimation of the error associated to the determined value.

Reporting for specific materials, systems and methods

We require information from authors about some types of materials, experimental systems and methods used in many studies. Here, indicate whether each material, system or method listed is relevant to your study. If you are not sure if a list item applies to your research, read the appropriate section before selecting a response.

Materials & experimental systems

n/a	Involved in the study
☒	☐ Antibodies
☒	☐ Eukaryotic cell lines
☒	☐ Palaeontology and archaeology
☒	☐ Animals and other organisms
☒	☐ Clinical data
☒	☐ Dual use research of concern
☒	☐ Plants

Methods

n/a	Involved in the study
☒	☐ ChIP-seq
☒	☐ Flow cytometry
☒	☐ MRI-based neuroimaging

Plants

Seed stocks

Report on the source of all seed stocks or other plant material used. If applicable, state the seed stock centre and catalogue number. If plant specimens were collected from the field, describe the collection location, date and sampling procedures.

Novel plant genotypes

Describe the methods by which all novel plant genotypes were produced. This includes those generated by transgenic approaches, gene editing, chemical/radiation-based mutagenesis and hybridization. For transgenic lines, describe the transformation method, the number of independent lines analyzed and the generation upon which experiments were performed. For gene-edited lines, describe the editor used, the endogenous sequence targeted for editing, the targeting guide RNA sequence (if applicable) and how the editor was applied.

Authentication

Describe any authentication procedures for each seed stock used or novel genotype generated. Describe any experiments used to assess the effect of a mutation and, where applicable, how potential secondary effects (e.g. second site T-DNA insertions, mosiacism, off-target gene editing) were examined.
